# Advancements in drug delivery methods for the treatment of brain disease

**DOI:** 10.3389/fvets.2022.1039745

**Published:** 2022-10-18

**Authors:** Brittanie Partridge, Allison Eardley, Brianna E. Morales, Sabrina N. Campelo, Melvin F. Lorenzo, Jason N. Mehta, Yukitaka Kani, Josefa K. Garcia Mora, Etse-Oghena Y. Campbell, Christopher B. Arena, Simon Platt, Akiva Mintz, Richard L. Shinn, Christopher G. Rylander, Waldemar Debinski, Rafael V. Davalos, John H. Rossmeisl

**Affiliations:** ^1^Veterinary and Comparative Neuro-Oncology Laboratory, Virginia-Maryland College of Veterinary Medicine, Virginia Tech, Blacksburg, VA, United States; ^2^Walker Department of Mechanical Engineering, University of Texas at Austin, Austin, TX, United States; ^3^Department of Biomedical Engineering and Mechanics, Virginia Tech, Blacksburg, VA, United States; ^4^Department of Small Animal Medicine and Surgery, University of Georgia, Athens, GA, United States; ^5^Department of Radiology, Columbia University Medical Center, New York, NY, United States; ^6^Department of Cancer Biology, Wake Forest Baptist Comprehensive Cancer Center, Winston-Salem, NC, United States

**Keywords:** blood-brain barrier, brain tumors, convection enhanced delivery, focused ultrasound, interstitial delivery, nanoparticles, pulsed electric fields

## Abstract

The blood-brain barrier (BBB) presents a formidable obstacle to the effective delivery of systemically administered pharmacological agents to the brain, with ~5% of candidate drugs capable of effectively penetrating the BBB. A variety of biomaterials and therapeutic delivery devices have recently been developed that facilitate drug delivery to the brain. These technologies have addressed many of the limitations imposed by the BBB by: (1) designing or modifying the physiochemical properties of therapeutic compounds to allow for transport across the BBB; (2) bypassing the BBB by administration of drugs *via* alternative routes; and (3) transiently disrupting the BBB (BBBD) using biophysical therapies. Here we specifically review colloidal drug carrier delivery systems, intranasal, intrathecal, and direct interstitial drug delivery methods, focused ultrasound BBBD, and pulsed electrical field induced BBBD, as well as the key features of BBB structure and function that are the mechanistic targets of these approaches. Each of these drug delivery technologies are illustrated in the context of their potential clinical applications and limitations in companion animals with naturally occurring intracranial diseases.

## Introduction

The brain is a complex, heterogenous, and extremely metabolically active organ. Under resting conditions, the brain receives a relatively large proportion of cardiac output, and is responsible for 20% of total body oxygen consumption despite only making up a small fraction of the total body mass of higher mammals (1). Neuronal synaptic activity is responsible for consuming 75–80% of energy produced in the brain (2). Precise regulation of the flux of biological substances into and out of the brain is fundamental to meeting the brain's dynamic metabolic demands, preserving physiologic neural signaling, and isolating the brain from exposure to blood-borne insults (3, 4).

The blood-brain barrier (BBB) plays a significant role in maintaining normal microenvironmental conditions in the brain by selectively allowing the influx of ions, essential nutrients and energy necessary for neural function while simultaneously buffering the interstitial fluid (IF) of the brain against fluctuations in the molecular composition of the plasma, exporting metabolic waste products, and limiting the entry of toxins and other exogenous compounds from the blood (3). Several structural properties of the BBB distinguish it from the systemic vasculature and contribute to its barrier functions including a high transendothelial electrical resistance (TEER), a low rate of molecular transport *via* transcytosis, and severely restricted paracellular permeability (3–5). Although these gate-keeping functions of the BBB have evolved to increase the efficiency of synaptic and neural network communications requisite to the performance of complex functions and behaviors, the selectivity of the BBB has been a major obstacle to the effective systemic delivery of therapeutic agents to the brain, with < 5% of small molecule pharmaceuticals effectively crossing the BBB (1, 4–6).

Here we review the structure and function of the BBB and recent technologic innovations that have been developed to enhance the delivery of therapeutic agents to the central nervous system (CNS) (6–8). These advancements, as well as their limitations, are illustrated through clinical applications in companion animals with naturally occurring neurological diseases. These delivery strategies will be covered in relation to the fundamental mechanisms by which they overcome the challenges posed by the BBB: (1) through the designing or modification the physiochemical properties of the therapeutic compound to facilitate transport across the BBB; (2) by bypassing the BBB *via* administration of drugs *via* non-conventional routes; and (3) by transiently disrupting the BBB (BBBD) using energy-based therapies (7, 8).

## Central nervous system (CNS) barriers to drug delivery

**Figure 1:**
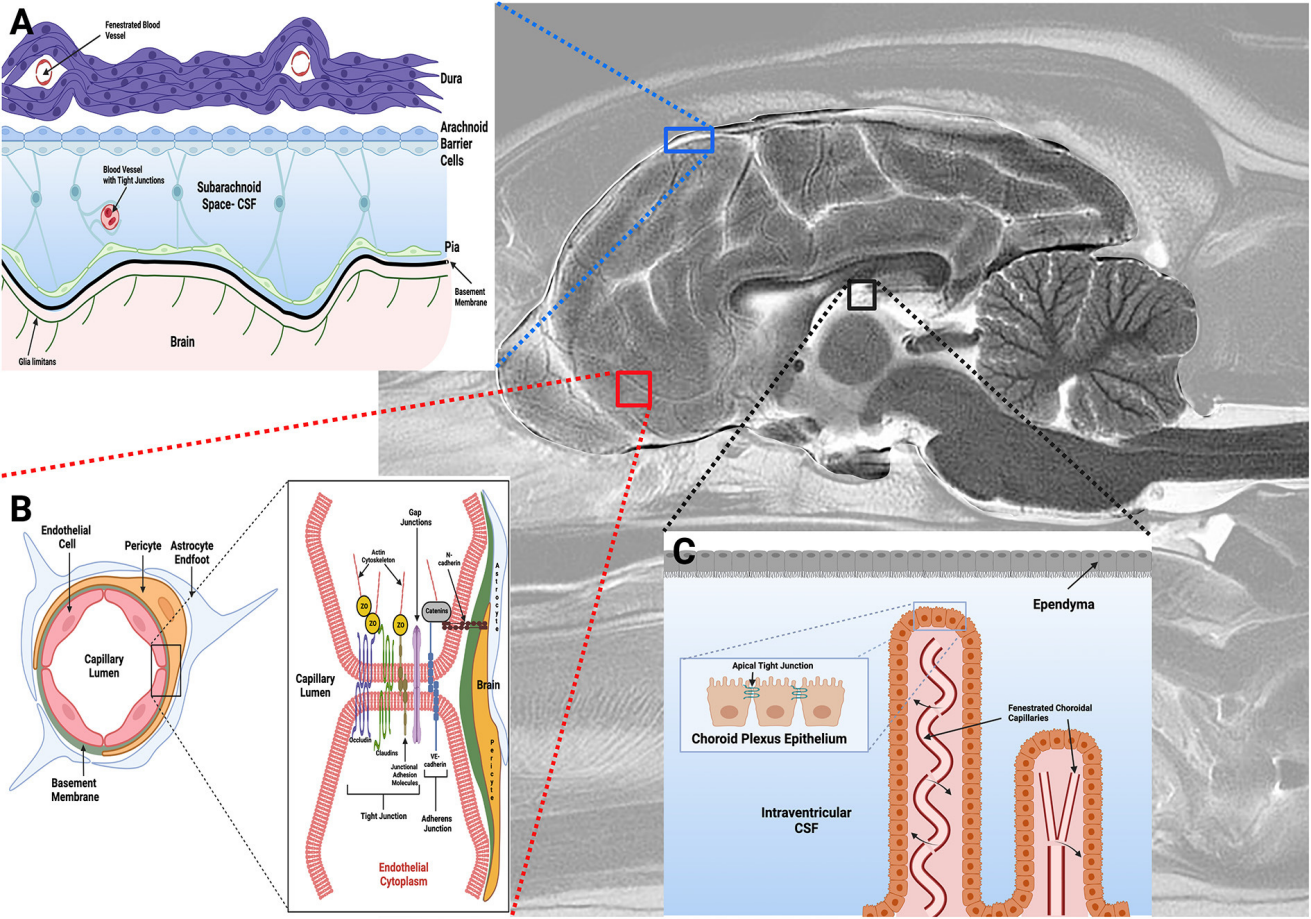
Barriers of the central nervous system. **(A)** The arachnoid barrier consists of arachnoid epithelial cells with tight junctions between adjacent cells, and forms a barrier between cerebrospinal fluid (CSF) in the subarachnoid space (SAS) and the more superficial dura mater. Blood vessels in the SAS have tight junctions with similar barrier characteristics as BECs in the BBB, while blood vessels within the dura are fenestrated. **(B)** The blood-brain barrier (BBB) exists at the level of the BECs, and is composed of a structural unit that also includes pericytes, astrocytic endfeet, and the basement membrane. Junctional complexes, including tight and adherens junctions (**B**, inset), between endothelial cells restrict paracellular movement of substances across the BBB. **(C)** The blood-CSF barrier is found in the choroid plexuses within the ventricular system of the brain, with apical tight junctions (**C**, inset) between choroid plexus epithelial (CPE) cells. The CPE contains apical microvilli, which increase the surface area of exchange between the CPE epithelial and CSF. Choroidal capillaries are fenestrated and do not form a barrier (arrows). Figure created with https://BioRender.com.

There are three barrier systems that physically and functionally separate the extracellular fluid of the CNS from the blood (Figure 1): the arachnoid, blood-cerebrospinal fluid (BCSFB), and blood-brain barriers (BBB) (3, 5, 9). Each of these barriers participates in the maintenance of CNS homeostasis by regulating substance exchange with the blood by physical means, such as occurs *via* restriction of paracellular transport by tight junctions, through molecular trafficking by specific transport systems, as well as through the metabolism of substances as they traverse these barriers (3–5).

### Arachnoid and blood-cerebrospinal fluid barriers

The arachnoid barrier consists of an avascular sheet of arachnoid epithelial barrier cells linked by apical tight junctions that forms a barrier between the cerebrospinal fluid (CSF) in the subarachnoid space (SAS) and the overlying dura mater (Figure 1A) (3, 9). The overall contribution of the arachnoid layer to substance exchange between blood and CNS is minimal due to the avascularity of this barrier, and its relatively small surface area compared to the other barriers (3). The BCSFB (Figure 1C) is found in the choroid plexuses (CP) of the ventricular system of the brain. The CP are veil- or frond-like structures, consisting of a central stroma with a high density of fenestrated capillaries and immune cells, surrounded by a single layer of cuboidal epithelial cells with apical tight junctions that face the ventricles (5, 9, 10).

### Blood-brain barrier (BBB) structure and function

The final barrier is the BBB (Figure 1B), which is composed of phenotypically specialized brain endothelial cells (BECs), a capillary basement membrane (BM), pericytes, and astrocytic foot processes (Figure 1B) (3, 4). The meshlike capillary network of the brain is extremely dense, with a surface area ranging from 100–200 cm^2^/g brain tissue depending on the anatomic region (3, 4). Since the BBB serves as the major interface for substance exchange between the peripheral circulation and the brain, and is thus the primary barrier limiting drug delivery; it will be the focus of the review.

#### BBB-brain endothelial cells (BECs)

The barrier functions of the BBB are primarily due to features of BECs, which are distinctive from endothelial cells found in systemic vascular beds, and strictly regulate passage of substances into the brain. Mature mammalian BEC lack fenestrations and have limited luminal pinocytotic capability, which significantly restricts transcellular diffusion of molecules (4, 5). Passive diffusion across the BBB is generally limited to lipid-soluble molecules with molecular masses between 400–500 Da, surface areas < 80 Angstroms (2), and a tendency to form < 6 hydrogen bonds in water (6, 11).

Paracellular movement of hydrophilic molecules across the BBB is limited by the expression of junctional complexes between adjacent endothelial cells (Figure 1B). Junctional complexes consist of a network of interdigitated transmembrane (claudins, occludin and junctional adhesion molecules), cytoplasmic (zonula occludens), and cytoskeletal (actin) proteins (3–5, 11). Tight junctions (TJ) mechanically link the apical membranes of adjacent BECs, consist of claudins-1,−3,−5 and−12 and occludin proteins, and directly contribute to BBBs high TEER, which is a quantitative measure of the integrity of a cellular barrier (3, 5, 6). Zonula occludens proteins bind the intracellular domains of claudins and occludin to the actin cytoskeleton, providing structural support to the TJ. Junctional adhesion molecules (JAMs) are members of the immunoglobulin superfamily of proteins that are colocalized with TJ. JAMs have several functions including promoting BEC polarity and localization of zona occludens proteins at points of cell-to-cell contact, supporting TJ through interactions with cytoskeletal scaffolding proteins, and participating in leukocyte migration across the BBB (5). Adherens junctions (AJ) are also found at the basolateral membrane of BEC. Similar to TJ, AJ contain transmembrane proteins, cadherins, which are responsible for mediating cell-cell adhesions and TJ assembly, and catenins, which support cadherin association with cytoplasmic scaffolds and regulate intracellular signaling (12). In BECs, the principle transmembrane AJ protein is VE-cadherin.

**Figure 2:**
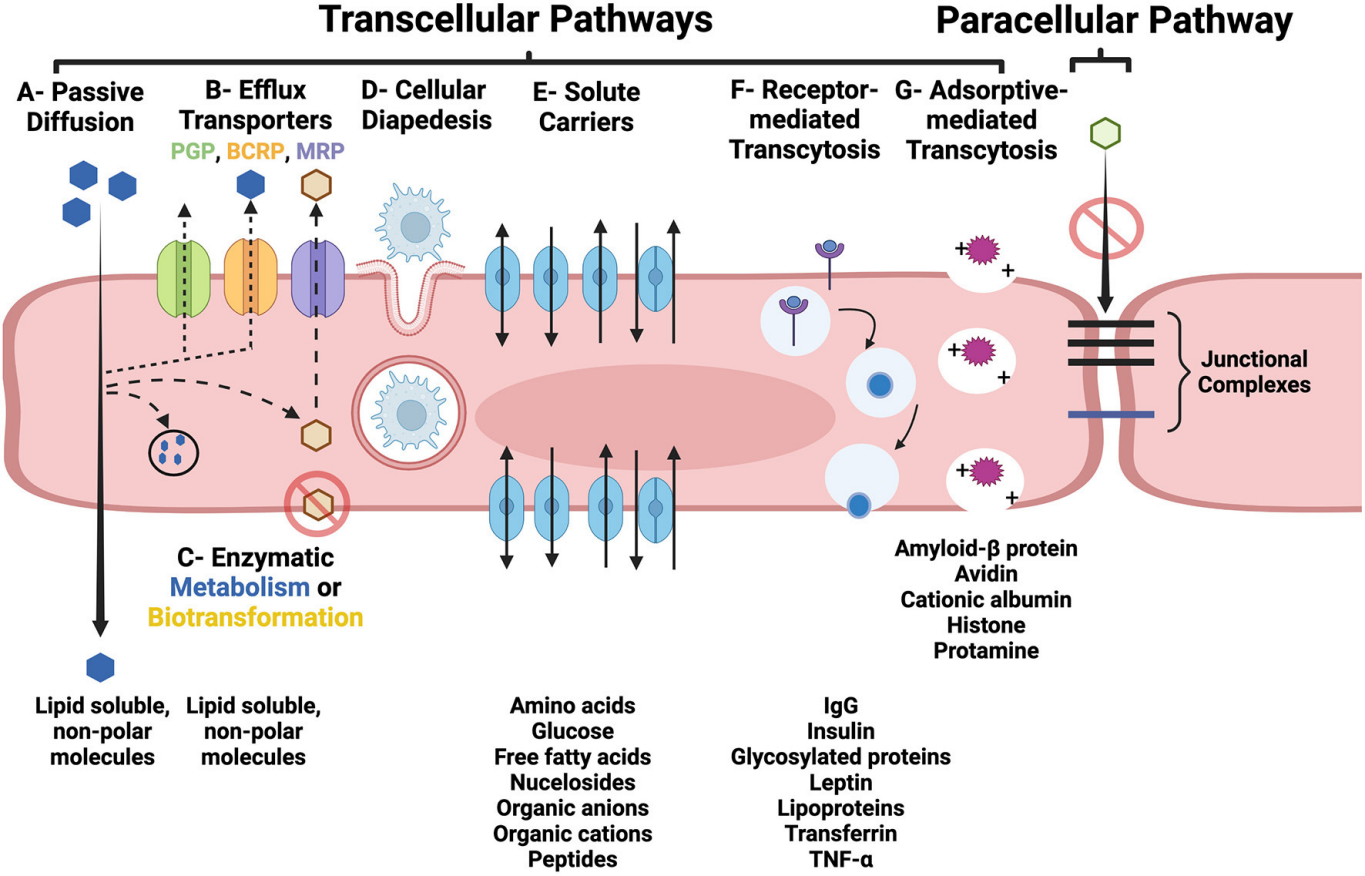
Transcellular **(A–G)** and paracellular pathways of transport across the BBB. **(A)** Lipid soluble, non-polar substances may passively diffuse through BEC. **(B)** Parent compounds and metabolites can also be subjected to removal from the BEC by active efflux transporters, such as PGP, BCRP, and MRP. **(C)** Lipid soluble molecules may be subjected to enzymatic metabolism or biotransformation which can prevent them from traversing the BEC and entering the interstitial fluid of the brain or facilitate their removal *via* efflux pumps. **(D)** Diapedesis of leukocytes through and intact BEC. **(E)** Carrier-mediated transport of many essential polar molecules and nutrients into the brain occurs *via* numerous specific solute carrier system. Receptor- (RMT; **F**) and adsorptive-mediated transcytosis (AMT; **G**) use vesicular based transport systems to move macromolecules across the BBB. RMT is initiated following ligand binding to a membrane receptor, and AMT is induced by interaction of positively charged macromolecules with the luminal BEC membrane. In the intact BBB, junctional complexes, including tight and adherens junctions, prohibit the paracellular transport of substances. Figure created with https://BioRender.com.

BECs also contain a multitude of active drug transporters in the ATP-binding cassette (*ABC*), and solute carriers (*SLC*) for organic anions, cations and peptide gene families (3, 4, 13). These transporters are integral membrane proteins that hydrolyze ATP to translocate substances across cellular membranes in all mammalian species, and are major determinants of drug distribution into, and elimination from, the brain. Efflux transporters in the *ABC* gene family, including P-glycoprotein (PGP), multidrug resistance protein (MRP), and breast cancer resistance protein (BCRP) have been recognized as major contributors to BBB function by actively transporting a wide variety of endogenous and xenobiotic lipophilic compounds out of BEC (Figure 2) (13). The vast array of substrates for which these specific efflux pumps have affinities for have been reviewed elsewhere (3, 13, 14).

Numerous neurotransmitter metabolizing enzymes are also expressed in the BBB (4). In addition, BEC have also been shown to contain several drug-inactivating enzyme systems, the most relevant to drug metabolism being members of the cytochrome P450, histamine *N*-methyltransferase (HNMT), and catechol *O*-methyl transferase (COMT) families (15). These enzyme systems can prevent substance entry into the brain *via* chemically inactivating or impeding drug passage across the BBB by structurally modifying drug polarity (15).

#### BBB- astrocytes

The terminal endfeet processes of astrocytes are in intimate association with the abluminal surface of BECs (Figure 1B). Molecules that regulate BBB ionic concentrations, such as Kir4.1 potassium and aquaporin-4 channels are highly expressed in astrocytic endfeet (3, 16). The close physical contact between astrocytes and BECs is critical for the development and maintenance of the BEC BBB-like phenotype (17). Non-neural tissues with fenestrated capillaries that were permeant to Evan's blue dye (EBD) under basal conditions became impermeant to EBD and developed an endothelial BBB-like phenotype following injection with astrocyte suspensions (18). The induction of BBB features in endothelial cells can also be triggered by exposure to soluble factors secreted from astrocytes, as bovine endothelial cells developed a higher TEER when cultured in astrocyte-conditioned media (19). The release of vascular endothelial growth factor, glial cell line-derived neurotrophic factor, basic fibroblast growth factor, and angiopoietin-1 from astrocytes has been shown to be critical to the formation of tight junctions in BEC, as well as to BBB repair and recovery in a variety of CNS diseases (3, 4, 20).

#### BBB-pericytes

Pericytes are important cellular constituents of capillaries and post capillary venules in the brain (21). Along with BECs, pericytes are enveloped in a continuous basement membrane that separates them from the astrocytic endfeet (Figure 1B). Pericytes are the most closely associated cell to BECs, being in direct contact with BECs *via* gap junctions as well as peg and socket type connections (21). Pericytes contribute to development and maintenance of the BBB by inducing downregulation of genes associated with pinocytic vesicle formation in developing BECs, producing components of the basement membrane, and through regulation of TJ proteins (21, 22). Pericyte co-culture with BECs will increase BBB TEER as well as efflux transporter function (23). Ongoing research suggests that cross-talk between astrocytes and pericytes, possibly through the platelet derived growth factor signaling pathway, is essential for the preservation of tight junctions (24).

#### BBB-basement membrane (BM)

The BM is a unique acellular layer of the extracellular matrix (ECM) that separates BECs from pericytes and the pericytes from underlying brain tissue (25). The BM is a highly organized, 50–100 nm thick proteinaceous sheet composed of collagen IV, laminin, nidogen and the heparin sulfate proteoglycan (perlecan), which are produced by BECs, pericytes, and astrocytes (26). The BM has many important functions, including providing structural support to the BBB, cell adhesion, participating in signal transduction, and regulating cell differentiation, migration, and survival. The contributions of the BM to BBB function are important. Loss of function mutations in BM components cause increased BBB permeability, intracerebral hemorrhage, and vascular and brain parenchymal malformations, although the mechanisms by which the BM participates in BBB maintenance and repair are incompletely understood (25, 27, 28).

### Transport across the BBB

There are two routes by which substances may cross the BBB: (1) the paracellular pathway, which involves passing between BECs, and (2) the transcellular pathway (transcytosis), which involves passing through the luminal side of the BEC membrane, traversing the BEC cytoplasm, and then passing across the basolateral side of the BEC into the interstitium of the brain (Figure 2) (3–5). Under physiologic conditions, BEC tight junctions prevent the paracellular molecular transport across the BBB. Transcytosis can occur through passive and active mechanisms. As reviewed in Section BBB-brain endothelial cells (BECs), passive diffusion across BECs is highly dependent on molecular characteristics, such as lipophilicity, electrical charge, and molecular weight (6, 11). As many essential polar molecules, such as glucose and amino acids, are incapable of diffusing across the BBB, these substances are transported in a carrier-mediated fashion using multiple specific solute carrier (SLC) systems. Carrier-mediated transport *via* SLCs may be: (1) limited to one direction, either into or out of the BEC; (2) bi-directional, with the direction of net transport being dictated by the concentration gradient; or (3) occur *via* exchange of substrates (4).

The transport of intact macromolecular complexes, poorly lipophilic compounds, or cells across the BBB involves active transport mechanisms *via* receptor-mediated transcytosis (RMT), adsorptive-mediated transcytosis (AMT), or cellular diapedesis (Figure 2) (3–5). In RMT, macromolecular ligands bind to specific receptors on BECs, which subsequently triggers endocytosis of the receptor and its bound ligand, packaging of this complex into a vesicle which is transported across the BEC cytoplasm, and finally binds with the basolateral membrane and is exocytosed. The dissociation of the ligand from its receptor may occur during vesicular transport or during the process of exocytosis. AMT involves interactions of cationic molecules with cell membrane binding sites which induces endocytosis and subsequent vesicular transcytosis (4, 29). Mononuclear cells are able to traverse the intact BBB through the BEC cytoplasm *via* diapedesis (30).

## Novel approaches to drug delivery to the brain

**Figure 3:**
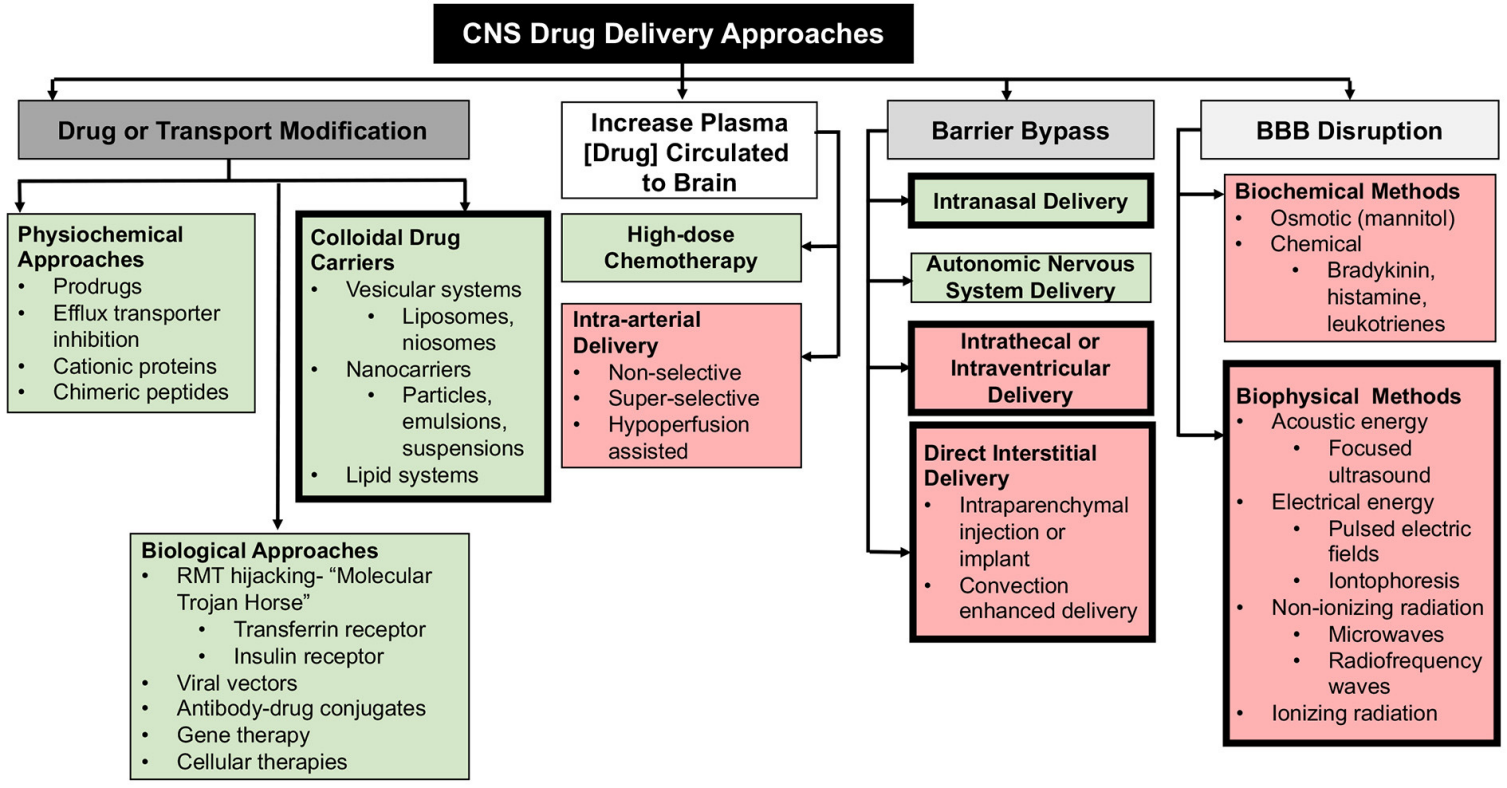
Mechanistic and methodologic summary of CNS drug delivery techniques. Non-invasive approaches are presented in green boxes, and invasive methods in red. Bold outlined boxes denote techniques that are covered in this review.

The complexities and difficulties associated with effective drug delivery to the brain is reflected in the sheer number of potential solutions that have been investigated (Figure 3). Mechanistic strategies to enhance drug delivery to the CNS broadly include: (1) design or modification of the physiochemical properties of substances to facilitate their transport into the CNS through transcellular pathways; (2) exposing the brain parenchyma to high concentrations of agents; (3) bypassing natural CNS barriers through locoregional drug delivery techniques; and (4) increasing paracellular transport through the BBB (7, 8, 11, 31). Comprehensive coverage of each of these methods is beyond the scope of this article, as these strategies have been reviewed in depth elsewhere (7, 9, 31–33). These drug delivery strategies are not mutually exclusive, as many are used in combination in an attempt to achieve the optimal drug delivery and thus desired therapeutic effect. In this review we focus on contemporary drug delivery strategies that have been developed, refined, and applied in companion animals with naturally occurring diseases within the last decade. Several other delivery approaches not specifically discussed here are under active clinical investigation in animals with neurological diseases. Early phase clinical trials using viral vectored and other gene therapy techniques are being conducted in pet dogs and cats with lysosomal storage diseases, degenerative myelopathy, brain tumors, and epilepsy. Studies evaluating the safety and feasibility of selective intra-arterial delivery of chemo- and radiotherapeutics in dogs with brain tumors are also being performed.

### Drug design or modification to facilitate transcellular transport

#### Colloidal drug carrier systems

Colloidal drug carrier systems (CDCS) are formulations in which microscopically dispersed drug particles are prepared in suspension or particulate formulations (34). CDCS can be classified by their physical form or functional profile, and there are numerous types including liposomes, emulsions, dendrimers, micelles, microparticles, minicells, and nanoparticles, among others. CDCS seek to improve the therapeutic index of drugs by increasing their efficacy or specificity and reducing toxicities. Potential advantages of CDCS as drug delivery platforms include increasing the bioavailability of drugs by increasing their diffusion through biological membranes, protection of drug payloads from hydrolysis or enzymatic degradation, allowing for design of sustained or controlled release drug profiles, and engineering for selective targeting (ligand-targeted CDCS) of cells or tissues (34, 35). In addition, in the setting of brain tumors, where segments of the tumor vasculature are structurally abnormal and leaky, CDCS may offer the additional benefit of an enhanced permeation-retention (EPR) effect, promoting accumulation of the therapeutic within the tumor parenchyma compared to normal tissues (33, 36).

The most commonly used CDCS in veterinary neurology consist of liposomal or lipid complexed (lipid-associated) amphotericin B preparations in the treatment of mycotic infections of the CNS (37, 38). Compared with conventional amphotericin B sodium deoxycholate, advantages of these CDCS formulations include reduced nephrotoxicity, fewer adverse infusion reactions, and better CNS penetration (37). There is insufficient available data regarding the relative efficacies of lipid-associated vs. conventional amphotericin B for the treatment of specific mycotic organisms in dogs and cats, as both of these formulations are often used as part of a multiagent anti-fungal treatment regimen (38).

Systemic administration of several therapeutic CDCS have been studied in dogs with naturally occurring brain tumors. One study exploited the overexpression of epidermal growth factor receptor (EGFR) that occurs in some canine brain tumors by administering weekly IV injections of bacterially derived minicells containing doxorubicin that target the EGFR receptor to 17 dogs with several types of presumptively diagnosed brain tumors (36, 39). Bacterial minicells are non-living, anucleate, non-growing, and non-dividing cells in the nano-size range (< 300 nm). Minicells are able to encapsulate a large number of biologically active agents including drug molecules, shRNAs, or siRNAs, and the minicell surface can be modified to incorporate bi-specific antibodies or other ligands (40). In this particular study, one arm of a bi-specific antibody was linked to the surface of the minicell, allowing the other arm to serve as a binding site for EGFR receptors presumptively expressed on the surface of the brain tumor cells (36). Accumulation of radiolabeled EGFR-targeted minicells within the parenchyma of tumors was demonstrated using single-photon emission computed tomography. Clinically, repeated IV administration of the minicells was very well–tolerated, with transient mild pyrexia and nausea being the most common adverse effects observed, and nearly 25% of dogs had objective anti-tumor responses evidenced by a significant reduction in tumor volume on follow-up MRI studies (36).

Another study was designed to evaluate the EPR of pegylated gold nanoparticles (GNP) in four dogs with spontaneous intracranial gliomas and meningiomas (41). This investigation utilized a treat-and-resect paradigm in which GNP were administered IV 1 day prior to surgical resection of the tumor, and the distribution and composition of the GNP within resected tumors were assessed microscopically, ultrastructurally, and chemically using Raman spectroscopy, scanning electron microscopy, and energy-dispersive X-ray spectroscopy (41). No serious adverse events attributable to the GNP infusion were observed, and the EPR of GNP was variable with heterogeneous distribution of the nanoparticles seen within different intratumoral compartments and tumor types. GNP were not identified in areas of normal brain tissue available for examination. This study illustrates that while GNP administered IV will extravasate into canine brain tumors, the inherent heterogeneity of the tumor microenvironmental influences on the potential drug biodistribution needs to be accounted for and assessed when performing clinical trials investigating CDCS formulations (41).

CDCS have also frequently been coupled with other drug delivery systems when treating diseases of the central nervous system. For example, liposomal and nanoparticle-based chemotherapeutics have been administered to the brain of dogs using interstitial delivery techniques. These combined approaches are presented in Section Barrier bypass delivery methods of this review.

### Barrier bypass delivery methods

#### Intranasal delivery

The nasal cavity offers a unique and advantageous option for delivering drugs directly and quickly to the brain due to its anatomical and physiological properties as well as its capacity of bypassing the BBB. It provides a large, highly vascular absorptive surface adjacent to the brain as well as a direct pathway for bloodstream absorption of drugs (32). Nasal drug delivery has numerous potential benefits over the IV, per rectum (PR), or per os (PO), routes, which may include non-invasive administration, more rapid onset of therapeutic effect, superior bioavailability by avoidance of hepatic first-pass metabolism, possibly increased CNS drug availability due to BBB bypass, and a lack of need to modify the parent drug to facilitate its delivery to the target (42). In veterinary clinical neurology, intranasal (IN) administration has been used for decades as a route to deliver therapeutics to the brain primarily in the context of anticonvulsant drug therapy for the treatment of seizures, but the transport mechanisms by which intranasally administered drugs reach the brain have only recently been elucidated (42–46).

There are several routes by which IN administered drugs may pass through the olfactory epithelium (32, 42, 45). The first is a route by which the drug is absorbed through the olfactory epithelium, which has intercellular clefts associated with its tight junctions, and then travels a paracellular route to the lamina propria. Another route involves transcellular passage of drugs through the supporting sustenacular cells of the nasal epithelium to the lamina propria. Once the drug reaches the lamina propria, it can subsequently enter the systemic vasculature *via* local blood vessels, be absorbed by lymphatics, or continue to travel a paracellular route into the perineural spaces surrounding the olfactory or trigeminal neurons to the subarachnoid space of the brain where it is further distributed by bulk fluid flow (45). The third route is an intracellular transport mechanism which involves endocytosis of the drug by olfactory sensory neurons or trigeminal nerve endings within the nasal mucosa, followed by axonal transport along the olfactory of trigeminal nerve pathways, where the drug is exocytosed into the synaptic clefts within the olfactory bulb or pontomedullary region, respectively. This transynaptic process is then repeated by intracranial olfactory or trigeminal neurons, which further distributes the drug to other brain regions (42, 45).

Delivery of benzodiazepines using the IN route has been demonstrated to be a safe and effective method for the treatment of emergent seizure presentations in both hospital and community settings when establishing timely vascular access is difficult or impossible (43, 44, 47). In the dog, IN midazolam is an established first-line intervention for in-hospital and at-home seizure treatment (47, 48). Despite being water-soluble, midazolam becomes lipid-soluble when at physiologic tissue pH, which enables it to cross the nasal mucosa and access the brain with rapid absorption (48). When the time required to establish IV access is factored into the seizure outcome endpoint, it has been shown that IN midazolam administration resulted in faster termination of seizures in dogs when compared to IV midazolam (43). In a multicenter controlled clinical study, the success rate of IN midazolam (70% response rate) was significantly superior in terminating status epilepticus in dogs when compared rectal diazepam (20% response rate) (44). IN administration also offers multiple practical advantages, including its ability to be performed by non-medically trained individuals, low risk of adverse effects or injury to the human administrator or animal when delivered using a mucosal atomization device, and its preference for home use by animal owners, particularly when compared to PR or buccal administration routes (43, 45, 47).

#### Intrathecal delivery

Intrathecal (IT) administration involves the injection of substances directly into cerebrospinal fluid (CSF) containing spaces within the CNS. Substances may be administered IT into the subarachnoid space by lumbar injection, cerebellomedullary cisternal injection, or by injection into the ventricular system (49). IT administration allows for bypass of the BBB and BCSFB and exploits the significantly smaller volume of distribution in the CSF space compared to plasma, which typically allows administration of a reduced drug dosage, simultaneously minimizing systemic toxicities and achieving high concentrations of therapeutic agent within the CNS (49, 50). Disadvantages associated with IT administration include a highly variable distribution of molecules within the CSF compartment that can be difficult to optimize, rapid clearance of hydrophilic agents as the CSF volume turns over 5–6 times/day in dogs, limited distribution of hydrophobic agents away from the injection site, and relatively poor penetration of macromolecular drugs into the brain parenchyma (50, 51). The use of CDCS may result in improvement of the distribution and brain penetration of IT administered drugs (50, 52). In small animals, there are additional drawbacks associated with IT administration including the practical requirements for anesthesia, and technical challenges associated with injecting agents into the lumbar subarachnoid space or ventricular system (53).

**Figure 4:**
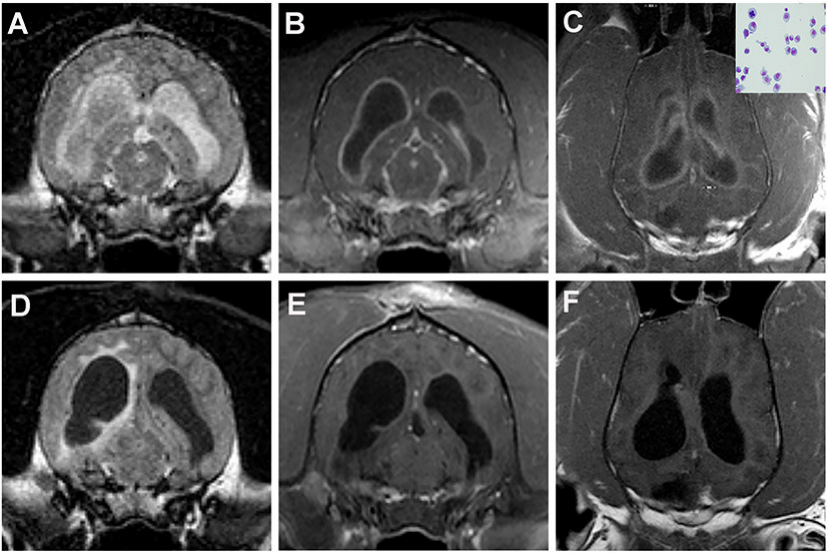
Intracranial plasma cell tumor in a dog treated with IT methotrexate. **(A)** The neoplasm manifests on imaging as diffuse meningitis and ventriculitis, as evidenced by the intra-and paraventricular FLAIR hyperintensities **(A)** and diffuse meningeal and ependymal enhancement on the post-contrast T1W images **(B,C)**. Neoplastic plasma cells can be observed in the CSF cytospin preparation (**C**, inset). One month after treatment, the previous hyperintense FLAIR CSF signal has resolved, although paraventricular hyperintensity persists **(D)**, and improvement in the contrast-enhancing lesion burden is noted **(E,F)**.

In humans, IT drug administration is most commonly used to provide analgesia or to treat spasticity and CNS neoplasms, and similar indications for IT therapy have been described in dogs and cats (52–55). Combined with local anesthetics, IT morphine is effective in providing analgesia to dogs undergoing pelvic limb orthopedic surgery, though pruritus and urinary retention are reported adverse effects (53, 56). A single IT morphine injection has reportedly been effective at managing postoperative pain and maintaining stable hemodynamics in elderly dogs with cancer undergoing major abdominal or thoracic surgery, while preventing many complications associated with parenteral opioid administration (54). Besides the performance of myelography, the most common therapeutic indications for IT drug administration related to veterinary neurology are the administration of cytosine arabinoside, methotrexate, or other agents for the treatment of hematologic malignancies with CNS involvement, primary brain tumors with leptomeningeal dissemination, or immune-mediated meningoencephalitides (Figure 4) (55–57). As both cytosine arabinoside and methotrexate are cell-cycle specific anti-cancer agents, IT administration is advantageous for treatment of CNS disease as it provides prolonged exposure of target cells to sufficient drug concentrations to facilitate cytotoxcitiy (55, 58). In people, the most common adverse event reported with IT cytosine arabinoside and methotrexate is a transient, self-limiting arachnoiditis (55, 58). Seizures, ascending myelopathic, and encephalopathic complications are reported much less frequently (55, 58). In a study investigating 120 dogs and cats with inflammatory or neoplastic CNS disease receiving a single IT injection of cytosine arabinoside alone or in combination with methotrexate, an adverse event was reported in only one dog, which experienced seizures following recovery from the procedure that responded to IV diazepam treatment (55).

To eliminate the need for and technical challenges associated with repeated IT injections, catheters can be inserted into the subarachnoid space or ventricular system, which are then connected to internal or external drug pumps and reservoirs to allow for continuous or intermittent IT drug delivery (59, 60). However, intrathecal catheterization for the purpose of drug delivery is not a commonly performed clinical procedure, being most often described in research setting using various small animal disease models (59, 60). Notably, the formation of pyogranulomatous masses in the region of the catheter tip is a recognized complication of chronic IT catheterization in the dog (60).

#### Interstitial delivery

**Figure 5:**
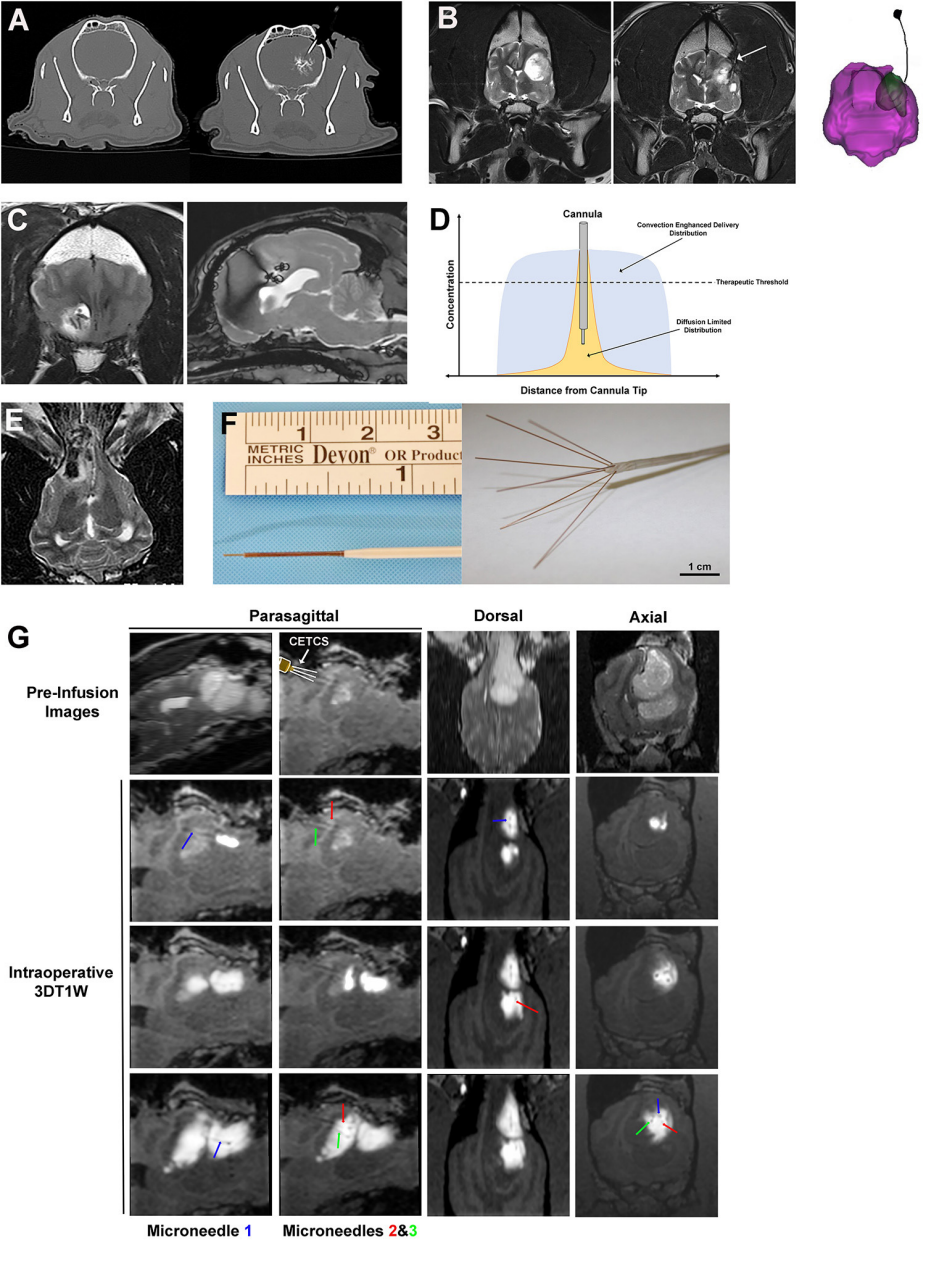
Interstitial approaches to drug delivery to the canine brain. **(A)** Interstitial bolus injection of nanoparticle-based temozolomide and iodinated contrast to the caudate nucleus of a dog, with unintended distribution of the infusate outside of the intended target region (right panel). **(B)** Pre-operative image (left panel) of high grade oligodendroglioma in the left frontoparietal lobes of the cerebrum, with a chronically implanted interstitial catheter for immunotherapy treatment visible in the tumor resection cavity (arrow, middle panel and far right panel). **(C)** Implanted, biodegradable microcylinders for sustained release chemotherapeutic delivery to a canine intracranial glioma. The microcylinders appear as discrete linear or round hypointensities within the T2W hyperintense tumor mass. **(D)** Schematic representation of infusate distributions achievable with interstitial delivery *via* diffusion (yellow) and convection (blue). **(E)** Iron-oxide nanoparticle CED in a dog with an oligodendroglioma in the fronto-olfactory region, with the infusate distribution appearing as the hypointense area within the T2W hyperintense tumor. **(F)** CED catheters; left panel demonstrates commercially available reflux preventing catheter with step-down features (ClearPoint Neuro, Solano Beach, CA, United States); right panel illustrates distal end of the CETCS system with microneedles arborized from the primary cannula. **(G)** MRI-guided CED infusion of a canine glioma with the CETCS. Individually deployed microneedles (blue, green, and red arrows can be visualized as thin, linear hypointensities extending from the primary cannula. The therapeutic was co-delivered with gadolinium-albumin, such that the infusate spatial distribution can be tracked in real time on the 3DT1W sequences.

While the IN and IT routes allow the BBB to bypassed to some extent, administration of drugs into the interstitium of the brain provides the most direct route of delivery. There are several technical approaches to direct interstitial delivery (Figure 5), which include parenchymal bolus injection, implantation of biocompatible and biodegradable materials, and convection enhanced delivery (CED). Advantages associated with interstitial delivery include the abilities to administer small molecule and macromolecular compounds and achieve high therapeutic drug concentrations in the brain while minimizing systemic drug exposures (61). Current disadvantages of interstitial delivery techniques are that they are all invasive and generally only suitable to treat loco-regional diseases of the brain, as none have been shown to be capable of efficient or safe global targeting of the brain parenchyma. There are also the practical difficulties associated with the need for long-term, repeated administration of appropriate drugs for many brain disorders, as well as the challenges controlling the spatial distribution of substances delivered to the interstitium of the brain, especially when bolus injections techniques are employed (Figure 5A). This can lead to reflux up the injection tract or exposure of non-target regions of the brain to therapeutics even when slow infusion rates are used (61).

##### Interstitial bolus injections

Interstitial bolus injections have been used for the delivery of immunotherapeutics to dogs with intracranial gliomas (62, 63). In one dose escalation study, a stimulator of interferon genes (STING) agonist was administered intratumorally every 4–6 weeks via Hamilton syringe to five dogs with intracranial gliomas (62). Activation of STING results in production of interferons and other pro-inflammatory cytokines, promoting infiltration of cytotoxic T-cells into the tumor, and reversal of immunosuppressive tumor microenvironments. One dog in this investigation developed signs of intractable intracranial hypertension following injection and was ultimately euthanized. Objective reductions in tumor size were demonstrated on follow-up MRI examinations in the dogs receiving the higher doses of the STING agonist (62). Another phase I trial performed in 21 dogs with intracranial gliomas utilized an implanted intraparenchymal catheter to deliver an oncolytic herpes virus vector (HSV-1) that was genetically engineered to express IL-12 into the resection bed following surgical debulking of the tumors (Figure 5B) (63). The utility of HSV-1 as a vector for CNS gene transfer has been previously demonstrated in the dog, as intracerebral injections of HSV-1 into normal dog brains did not result in any neuropathological lesions, latent viral DNA was detected in brain regions remote from the injection site, and human IL-12 will induce a systemic T-cell response in dogs (63, 64). No significant adverse events attributable to administration of HSV-1 or dose-limiting toxicities were observed in this study, and molecular and genomic analyses of a subset of treated canine tumors revealed tumoral transcriptional and peripheral blood cytokine signatures of anti-tumor immune pathway activation including interferon signaling, lymphoid and myeloid cell activation, and *T* and *B* cell immunity (64, 65).

##### Implantable biodegradable polymers

Numerous natural and synthetic polymers, including albumin, chitosan, gelatin, polycaprolactone, polylactic acid, and polylactic-co-glycolic acid (PLGA) have been used to formulate implantable biodegradable interstitial drug delivery systems for applications in the brain (66). When combined with a drug, the chemical composition of the polymer can be tuned to allow for a controlled release drug profile (66, 67). The concept of a sustained release interstitial drug delivery system has been evaluated in healthy dog brains and dogs with intracranial gliomas in which PLGA microcylinders containing temozolomide (TMZ) and gadolinium were implanted intracerebrally (Figure 5C) (68, 69). In these studies, the microcylinders were designed to have a 30 days degradation time, and were clearly visualized on post-implantation MRI scans, as was diffusion of the gadolinium into the surrounding brain parenchyma over time. There was mild and transient clinical morbidity associated with free-hand implantation of the microcylinders, suggesting that the polymer system and technique are feasible for the sustained interstitial delivery of TMZ (69).

An additional limitation of interstitial bolus injections and implantable biomaterials is that the drug's distribution within the interstitium following injection or implantation is diffusion dependent (61, 68, 70). An investigation of the brain penetration of a carmustine impregnated polymer indicated that drug distribution occurred up to 5 mm from implant site on the first day after implantation, but was reduced to 1 mm away from the implant after the third day (70). The superior distribution into the brain parenchyma observed acutely after implantation or injection is postulated to occur due to tissue convection secondary to procedurally-induced vasogenic edema, which is a generally a transient process that resolves within a few days (70). These studies indicate that while interstitial injections and biodegradable implants are capable of producing very high local drug concentrations in the brain, their distribution into the brain parenchyma remote form the treatment site will be dictated by the physiochemical properties of the agent and will generally be limited to a few millimeters due their dependence on diffusion in conjunction with their rapid clearance from the brain (Figure 5D) (66–71).

##### Convection enhanced delivery (CED)

Another method of direct, interstitial delivery involves the placement of catheters into the brain and subsequent infusion of agents under a constant hydrostatic pressure gradient into the extracellular space of the brain, while displacing the extracellular fluid, a procedure that is termed convection enhanced delivery (CED) (61). The CED technique produces bulk fluid flow by convection, which allows for a more homogenous distribution of the infusate over a significantly larger volume of tissue than what can be achieved by simple diffusion (Figures 5D,E). Diffusion relies on a concentration gradient and operates according to Fick's law, which essentially states that molar flux is proportional to the concentration gradient multiplied by a tissue diffusivity coefficient. As the tissue diffusivity coefficient is dependent on molecular weight, macromolecular therapeutic agents take a long time to diffuse and require high concentrations to drive their flow (72). In the case of CED, a pressure gradient distributes the infusate by bulk flow, which is described by Darcy's law. Darcy's law states that the velocity of the molecule is directly proportional to the applied pressure gradient and the hydraulic conductivity. Thus a distinct theoretical advantage of CED is its ability to distribute drugs uniformly regardless of molecular weight, although the surface properties of the infusate have been shown to be critical determinants of interstitial spread of macromolecules molecules (73). However, there is a ceiling at which the distribution of large molecules will be limited by the capacity of the extracellular space within the brain.

The cumulative experience with CED over the past 25 years in animals and humans has clearly demonstrated that there are specific challenges associated with the technique (61, 72, 74). A number of limitations have been observed including the technical specifications and performances of the type(s) of catheter used for CED, modeling and achieving uniform infusate distributions within heterogeneous normal and pathological brain tissues, and optimizing the numerous variables associated with the infusion and the specific agent being infused, such as flow rate, catheter insertion and withdrawal rates, and the volume of distribution to volume of infusion ratio (V_d_:V_i_) (72, 74–76). Considerable advancements in novel or refined procedures and technologies that address the current limitations of CED have been evaluated in dogs with naturally occurring brain diseases.

Improvements in catheter design (Figure 5F) have been fundamental to the evolution of CED as therapeutic platform. The use of small diameter reflux preventing catheters (RPC) with step down features characterized by sequentially smaller shaft diameters along the working length of the catheter with the smallest opening at its distal tip of the catheter have been shown to produce balanced forward infusate dispersions and reduced clogging and infusate reflux along the catheter shaft, even at high infusion rates, relative to other types of catheters that have been previously used for CED (61, 74, 77). Additionally, a multiport CED catheter that incorporates a step down feature has been created with the intent of providing the treating clinician with a delivery system with greater latitude to alter the infusion configuration and expand the therapeutic capabilities based on clinical needs, and is called the convection-enhanced thermotherapy catheter system (CETCS; Figures 5F,G) (78). The CETCS primary cannula has multiple infusion ports, with each infusion port containing a fused-silica microneedle that is able to be individually deployed a variable distance away from the primary cannula. The CECTS provides several advantages over utilizing multiple single-port catheters, as the primary cannula requires a singular insertion path, with each microneedle being 10-fold smaller in diameter than conventional catheters, thus mitigating risks associated with the passage of multiple larger bore catheters, and the arborizing features of the microneedles can enhance the V_d_ of the infusate to the target region (79). Each microneedle in the CETCS system is also capable of co-delivering laser energy and an infusate, thus expanding the therapeutic capabilities of the system to include laser interstitial thermotherapy and optogenetic therapy (80). Further the photothermal capabilities of the CETCS can be exploited to cause mild, sub-lethal hyperthermia in the treatment field, which has been shown to facilitate volumetric dispersal of infusates during CED (81). The CETCS system has been used successfully to deliver molecularly targeted therapeutics to canine brain tumors (Figure 5G) (57, 82).

Studies in dogs with brain tumors have also reinforced the need for and illustrated the value of incorporating real-time MRI imaging into CED treatments (61, 74, 77, 82–84). MRI monitoring of CED procedures is generally performed by co-infusion of, or conjugation of the therapeutic agent to a superparamagnetic iron oxide-based (Figure 5E) or paramagnetic gadolinium-based (Figure 5G) contrast agent, with the contrast agent distribution serving as an imaging surrogate for distribution of the therapeutic (74, 84). Real-time imaging of CED has been shown to be essential for confirmation of catheter positioning, assessment of adverse effects associated with catheter placement and removal, quantitative evaluation of the infusate V_d_ and target coverage, and assessment of therapeutic efficacy as a function of target coverage (74, 77, 84). In addition, real-time imaging monitoring of the infusion provides an opportunity to assess unintended infusion complications, such as infusate reflux, or leakage into the ventricles or subarachnoid space, and promptly mitigate them to allow for continued target coverage (72, 74, 77). Imaging is also important for the assessment of adverse events associated with infusions that result in coverage of non-targeted brain regions (74).

The highly variable results achieved in early pre-clinical CED studies with respect to the quality and consistency of infusions highlight the numerous variables that contribute to the ultimate outcome of the infusion. These include the catheter location, type, and number; infusion rate, and volume; as well as physiochemical factors related to brain tissue viscoelastic characteristics, blood flow, and drug properties and pharmacodynamics. Significant efforts are being devoted to developing patient- and disease-specific computational models that account for these variables and can predict the drug distribution within the brain (72, 75). Some of these modeling techniques have been developed and applied in dogs (74, 85). An inverse shape-fitting therapeutic planning technique was utilized to identify the optimal position of CED catheters in dogs with intracranial gliomas (74). As catheter position is a major factor related to the V_d_ of infusates, use of this therapeutic planning technique resulted in quantifiable improvements in target coverage compared to a prior study in canine gliomas using the same type of catheter in which the neurosurgeon positioned the catheters based on clinical judgement (74, 77).

A variety of therapeutic agents have been delivered to canine brain tumors, the majority of which have been gliomas, *via* CED, including liposomal CPT-11 and nanoparticle-based temozolomide chemotherapies, targeted nanoparticular agents (EGFR-antibody bioconjugated nanoparticles), and recombinant bacterial cytotoxins and chemotherapeutic conjugates targeting the EphA2, EphA3, EphB2, and IL-13RA2 receptors that are overexpressed in canine gliomas (74, 77, 82–84). These early phase studies have illustrated the feasibility and safety of image-guided CED procedures in the dog brain, as well as providing preliminary evidence of efficacy of these investigational agents. Although studies using CED have only been examined to date in the context of canine brain cancer, this drug delivery platform is actively being investigated for use in neurodegenerative disorders, epilepsy, and stroke (72).

### Biophysical methods of transient BBB disruption (BBBD)

#### Focused ultrasound BBBD (FUS-BBBD)

**Figure 6:**
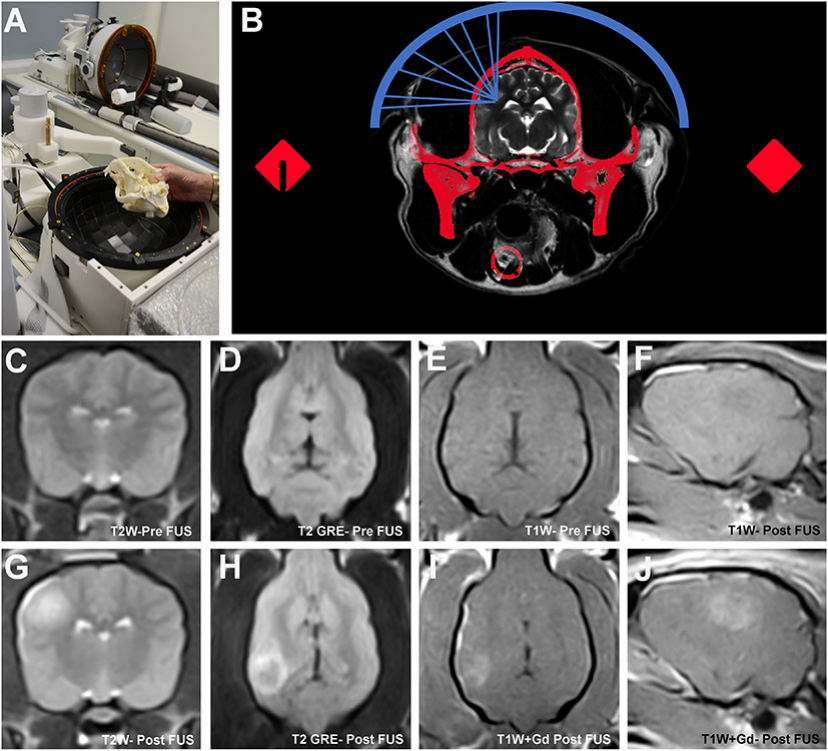
Transcranial magnetic resonance image guided focused ultrasound BBBD (MRg-FUS-BBBD) in the dog. Representative canine skull placed into a hemispherical phase-array transducer **(A)**, which contains multiple ultrasound elements for focusing of the ultrasound waves. The dog's head is immobilized using a headframe and coupled to the transducer using a malleable bladder filled with cooled, degassed water to facilitate acoustic coupling and reduce heating. Schematic **(B)** demonstrating non-invasive focusing of the multiple ultrasound beams (blue) through the skull (red) to the intended target. A computed tomographic (CT) image of the skull is obtained prior to treatment that is that is subsequently registered to the MRI. Information from the CT is used to correct for beam aberrations due to inhomogeneities in the skull, and accurately focus the beams on the target. Pre-sonication transverse T2W **(C)**, dorsal T2 GRE **(D)**, dorsal T1W **(E)** and parasagittal T1W **(F)** MR images illustrating normal canine brain signal characteristics and morphology. MRg-FUS BBBD manifests as a focal region of T2W hyperintensity **(G,H)** representing edema, which correspond with a region of contrast uptake on T1W images following gadolinium (Gd) administration **(I,J)** in sonicated locations.

Focused ultrasound (FUS) is a non-invasive technology in which propagated acoustic waves are used to achieve the intended therapeutic effects. In contrast to conventional diagnostic ultrasound in which echoes generated at tissue interfaces are acquired for imaging purposes, therapeutic FUS utilizes concave transducers that deliver the majority of their power to a single geometric focal point in order to induce thermal or mechanical effects within the target tissue (Figure 6) (86). The tissue level effects of sonication, and thus therapeutic applications, are generally related to the intensity of the acoustic energy delivered (Table 1) (86, 87). However, as a several key variables, including the fundamental frequency, pulse repetition frequency, duty cycle, sonication duration, and intensity, can be adjusted in a therapeutic ultrasound protocol, the entire range of biological effects that can be induced or manipulated in the FUS parameter space is currently unknown (86, 87). In clinical settings, FUS protocols that are labeled as “low intensity” typically employ ultrasound intensities at or below those used in diagnostic ultrasound imaging studies.

**Table 1:** Focused ultrasound treatment protocols and indications in the brain.

**FUS protocol**	**Ultrasound central frequency range**	**Tissue effects**	**Potential indications (87)**
High intensity focused ultrasound (HIFU)	20 kHz−200 MHz	Thermally mediated coagulative necrosis	Tumor ablation; thalamotomy for essential tremor or neuropathic pain
Low intensity focused ultrasound (LIFU) with microbubbles	220 kHz−800 kHz	Mechanically mediated transient opening of the BBB	Drug, cell, or gene delivery to the brain
LIFU	220 kHz−1.9 MHz	Suspected mechanical interference with voltage-dependent neuronal ion channels	Neuromodulation- activation of motor responses, suppression of epileptiform activity

The ability of FUS to induce BBBD and thus facilitate drug delivery to the CNS has been recognized for nearly 70 years. Early *in vivo* experiments with FUS performed using high intensity tissue ablation protocols demonstrated BBBD that was associated with significant collateral damage of the neuropil (88). The mechanical cavitation induced by high intensity FUS was the proposed mechanism of BBBD, but the large amount of energy required to achieve cavitation resulted in the undesirable adverse effects of neuronal damage (89, 90). Further investigations confirmed that sonication using lower frequency ultrasound pulses in the presence of circulating ultrasound contrast agents could open the BBB without causing unintended damage to the neural parenchyma, and BBBD could monitored in near real time using MRI. These observations gave rise to the contemporary field of transcranial magnetic resonance imaging guided FUS BBBD (MRg-FUS BBBD; Figure 6), which has subsequently been shown in pre-clinical animal experiments and human clinical trials to safely cause precise, accurate, and transient BBBD with high temporal and spatial specificity (87, 90–93).

Intravenously administered microbubbles are the ultrasound contrast agents most often utilized in MRg-FUS BBBD procedures. Microbubble formulations are a class of ultrasound contrast agents ranging from 1.5–5 μm in diameter that consist of a perfluorocarbon gas core coated by a lipid or albumin shell, and some microbubble products are approved for human use in diagnostic ultrasound imaging vascular applications. Following IV administration and in the presence of an ultrasound field, circulating microbubbles will oscillate and generate cavitations which produce several effects that act on the endothelium and ultimately lead to BBBD. The presence of the intravascular microbubbles allows for a reduction in the applied ultrasound intensity necessary to achieve BBBD, largely restricts the induced bioeffects to the vasculature, and thus mitigates adverse effects to extravascular tissues (92). FUS-BBBD results from three mechanistic phenomenon acting on the cerebral vasculature: disruption of tight junctions, stimulation of caveolar transcytosis, and sonoporation of the vascular endothelium (87, 91, 92). Therapeutic agents and as large as 2000kDa and intact cells have been shown to be able to penetrate the brain following FUS-BBBD, and the BBB permeability remains increased for several hours immediately after treatment (87, 92).

Based on previous investigations in transgenic rodent models of Alzheimer's disease which found that FUS-BBBD performed in the absence of additional therapeutics effectively reduced the beta-amyloid plaque burden in treated regions and improved spatial memory function, a FUS-BBBD study was performed in geriatric dogs with naturally occurring amyloid pathology in the brain (94, 95). MRg-FUS-BBBD was performed using IV microbubbles and a custom MRI-compatible system in one cerebral hemisphere in 10 aged dogs that were divided into two groups that received either a single treatment or four weekly treatments (95). BBBD was quantitatively confirmed on immediate post-treatment post-contrast MRI images in all dogs, and no dog experienced a neurological adverse event following treatment. One-week post-treatment follow-up brain MRI exams revealed that the BBB was intact in all animals with no imaging evidence of damage to the brain tissue in treated regions. Although no statistically significant differences were observed in beta-amyloid load between treated and untreated hemispheres in either group, this study demonstrated the feasibility and safety of repeated, large-volume FUS-BBBD in dogs (95).

Current disadvantages associated FUS-BBBD include the relatively short therapeutic window associated with BBBD, which would likely require repeated treatment sessions to manage diseases requiring chronic drug administration and the current lack of commercially available FUS systems designed for companion animal use. Although human MRg-FUS-BBBD systems are commercially available and have been used in dogs (Figure 6), they are extremely costly, require hardware adaptations for immobilization and acoustic coupling for use in animals, and require additional therapeutic planning procedures to account for beam attenuation associated with the significant intra- and inter-animal variability in skull geometry and density found in dogs and cats.

#### Pulsed electrical field induced BBBD (PEF-BBBD)

Pulsed electrical field (PEF) therapies use electrical energy that is intermittently delivered (i.e., pulsed) to tissues during a treatment session to cause various biophysical effects. The type of tissue changes induced are dependent on both the applied electrical field, which can be influenced by the pulse amplitude, shape, number, and length, as well the specific geometric and physiochemical characteristics of the tissue being treated. Electroporation is one type of PEF technique in which nanopores form in cell membranes following exposure to electric fields of sufficient amplitude and duration (96, 97). These nanopores permeabilize the membrane, and this permeabilization effect can take two forms depending on the applied electrical field: reversible electroporation, in which the permeabilization is transient and membrane integrity is quickly restored, and irreversible electroporation (IRE), in which the permeabilization disrupts cellular homeostasis and leads to cell death (96, 97). Reversible electroporation is used clinically to facilitate the targeted intracellular delivery of DNA, drugs, and chemotherapeutics, whereas to date, IRE has principally been used as non-thermal method of solid tumor ablation (96–98).

The IRE procedure requires the placement of minimally invasive electrodes within target tissue in order to deliver short duration (~10–100μs) PEFs. Application of electric fields that exceed 500 V/cm result in subsequent cell death of the target tissue characterized by sharply delineated region of necrosis in the treated area without collateral damage to critical neighboring structures, such as blood vessels and ducts (96–99). Clinical trials in animals and human with several types of cancers, including brain tumors in dogs, have demonstrated the safety and feasibility of IRE for tumor ablation (96–99).

When IRE pulses are applied in normal and neoplastic brain tissues, the treatment results in a central core of ablated tissue surrounded by a penumbra of voltage-dependent reversible electroporation and transient BBBD (99–101). Thus, in the context of therapeutic management of CNS malignancies, IRE is a potentially advantageous technique in that a single treatment session allows for ablation of the macroscopic tumor mass, while simultaneously transiently permeabilizing the BBB in a zone of brain tissue surrounding the tumor mass which can be exploited to deliver drugs to infiltrating tumor cells that often extend into the neural parenchyma beyond the gross tumor margin (98–100, 102). Some disadvantages associated with IRE include pulse-induced muscle contractions and the potential for treatment associated cardiac arrhythmias, which necessitate use of neuroparalytics and cardiac synchronization devices during pulse delivery (97, 99). Further it has been shown that in heterogeneous tissues, such as the brain and many types of tumors, the electric field distribution may be distorted, which can negatively impact the intended therapeutic effects of the applied IRE pulses (96, 97, 99).

**Figure 7:**
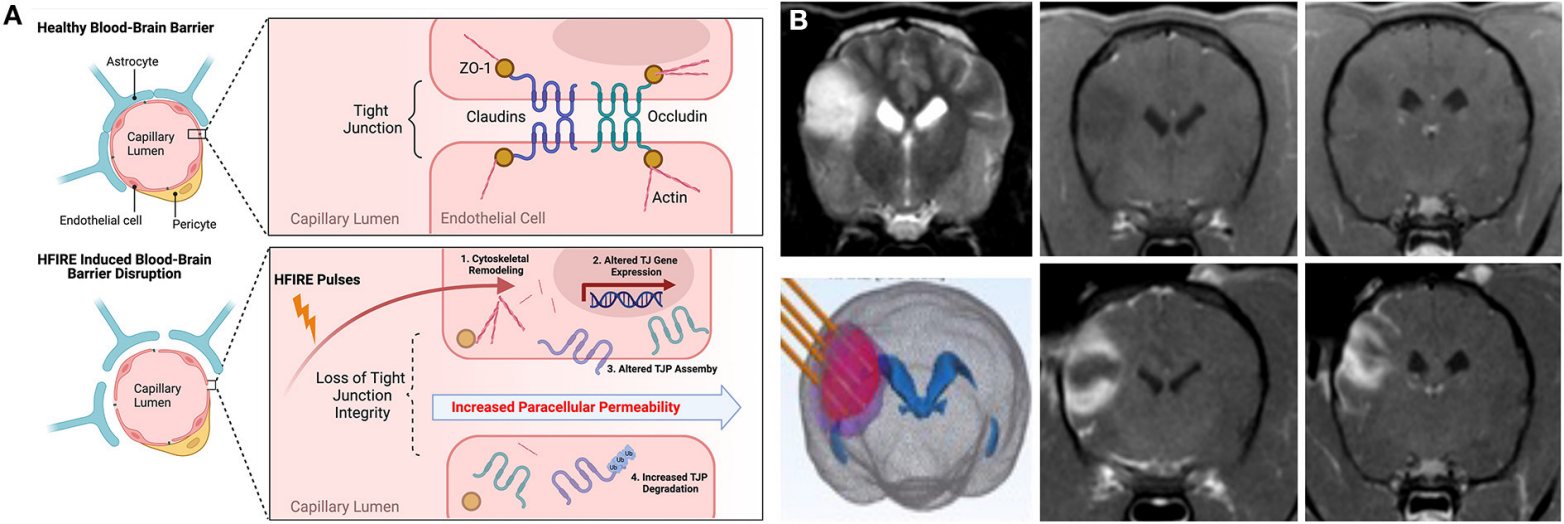
HFIRE-induced BBBD. **(A)** Schematic representation of mechanisms of HFIRE-induced BBBD (11) TJP, tight junction proteins; UB, ubiquitinated protein. Figure panel created with https://BioRender.com. **(B)** HFIRE induced BBBD in a non-enhancing canine oligodendroglioma. Top row demonstrating pre-treatment T2W (left panel) and T1W post-contrast images (middle and right panels) of the tumor with no abnormal areas of contrast-enhancement. HFIRE treatment (bottom row) planning image with stereotactic electrodes inserted into the tumor (left panel), and post-contrast T1W images (middle and right panels) demonstrating peripheral ring of gadolinium enhancement surrounding the tumor following HFIRE treatment.

High-frequency irreversible electroporation (HFIRE) therapy evolved from IRE and was developed to overcome some of the limitations observed with IRE pulses. HFIRE differs from IRE in that it utilizes ultrashort (~0.5–10μs) bursts of bipolar PEF to induce tissue bioeffects (103). Pulse paradigms used in HFIRE have been shown to mitigate treatment induced muscle tetany, create a more homogeneous electrical field distribution in complex tissues, and achieve BBBD in a region surrounding the treatment zone similar to IRE, while still achieving sharply demarcated, non-thermal ablations (103, 104). Application of HFIRE pulses at lower electrical field strengths can be used to transiently open the BBB without inducing any damage to the treated brain tissue for up to 72 h after treatment (11). HFIRE induced BBBD has been shown to result from increasing BBB paracellular permeability, which occurs secondary to BEC cytoskeletal remodeling, disruption of tight-junction integrity, and increased tight junction protein degradation (Figure 7A) (11).

HFIRE therapy has been used to ablate numerous naturally occurring canine tumors, including brain tumors (97, 105). In the context of treating canine gliomas with HFIRE, application of ablative pulses will also result in a peripheral zone of BBBD (Figure 7B) surrounding the ablated core that can be used to deliver therapeutic agents to the tumor margin. Limitations of IRE- and HFIRE-BBBD are similar to those discussed for FUS-BBBD (Section Focused ultrasound BBBD (FUS–BBBD)) with the notable exceptions of the invasiveness associated with electrode placement in the brain required to induce BBBD with IRE and HFIRE, and the need for development of patient-specific computational treatment plans to achieve safe and effective ablations (97, 99, 105, 106). However, proof-of-concept studies have recently been conducted in rodents illustrating that non-invasive, transcranial transient BBBD can be achieved using low-electrical field PEF (107).

## Conclusions

The BBB performs essential functions in protecting the brain from toxins and maintaining CNS homeostasis, but its structural and functional gatekeeping roles require the design of new drugs and innovative treatment strategies for treating diseases of the brain. Considering the significant global contribution of neurological disorders to morbidity and death in companion animals, as well as the veterinary community's continuously evolving understanding of the pathogenesis and genetic causes for many neurological diseases, we anticipate the clinical need for improved CNS drug delivery will increase with time and in parallel with the development of new therapeutics (108–110). This review highlights some of the recent advances that may expand the repertoire available to veterinarians to deliver drugs to the CNS. It is important to note that with the exceptions of intranasal and intrathecal delivery techniques, the remainder of the methods discussed here are still considered investigational approaches in animals and humans that do not have currently approved clinical uses (Table 2). Through the design of novel therapeutic compounds or combining new drugs with non-conventional routes of administration, such as *via* direct interstitial delivery or transient modulation of BBB permeability, the feasibility of several new strategies to overcome the BBB have been demonstrated in animals with naturally occurring brain diseases, paving the way for broader clinical applications of these techniques.

**Table 2:** Current, comparative regulatory landscape of cns drug delivery techniques and agents for neurological disease.

**Drug delivery** **technique**	**Examples of regulated drugs or devices/indications in humans**	**Investigational uses/** **indications in humans**	**Examples of unapproved or off-label routine clinical use in animals**	**Investigational uses/indications in animals**	**Pre-clinical evidence of Efficacy**
Intranasal					
• Drugs	Diazepam (Valtoco)* • Seizures Esketamine (Spravato)* • Depression	Many formulations • Narcolepsy • Neurodegenerative • Neuropathic pain • Stroke	Diazepam (46–48), Midazolam (43, 44) Lorazepam • Seizures	Many off-label and investigational agents • Behavioral • Epilepsy • Neurodegenerative	Yes
Intrathecal/intraventricular					
• Devices • Drugs	SmartFlow^®^ cannula^†^ methotrexate* Cytosine arabinoside* • Leukemia Baclofen* • Spasticity/dystonia	Many commercial and prototype catheters (67) Many off-label and investigational agents (67) • Brain tumors • Neurodegenerative • Neuropathic pain • Stroke	Many commercial catheters methotrexate (55) Cytosine arabinoside (55) • CNS malignancies, MUE Baclofen • Spasticity/dystonia	Many commercial and prototype catheters (60) Many investigational agents (108) • Brain tumors • Neurodegenerative • Neuropathic pain	Yes Yes
Interstitial drug delivery					
• Biodegradable implants • Convection enhanced delivery • Devices • Drugs	Gliadel (BCNU) wafer* • Glioblastoma Cleveland multiport catheter∧ None	Many investigational agents (33, 66) • Brain tumors • Stroke • Traumatic brain injury Many commercial and prototype devices (75), Many off-label and investigational agents • Brain tumors • Neurodegenerative • Stroke	None None None	Temozolomide (68, 69) • Glioma Many commercial and prototype devices (61–63, 74) Temozolomide, Topotecan Irinotecan, investigational agents (74, 77, 82–84) • Brain tumors	Yes Yes Yes
Focused ultrasound technologies					
• Devices • Drugs	Exablate neuro^•^ None	Several investigational devices (92) • Brain metastases • Malignant glioma • Neurodegenerative Pembrolizumab, investigational agents	None None	Several investigational devices (92, 93, 95) • Cognitive dysfunction • Brain tumors Several investigational agents (93)	Yes Yes
Pulsed electrical field technologies					
• Devices • Drugs	Nanoknife^•^ None	Investigational device (98) • Electrochemotherapy for brain metastases Bleomycin	None None	Several investigational devices (93, 97, 99) • Electrochemotherapy for glioma Bleomycin, doxorubicin, investigational agents	Yes Yes

*Approved for use by the United States Food and Drug Administration (FDA).

^†^Device has FDA 510K clearance for injection of cytosine arabinoside into the ventricle and is CE marked in Europe for delivery of approved fluids into the brain.

^∧^Device has FDA 510K clearance as a therapeutic delivery device for the brain.

^®^Device has FDA approval to treat Parkinson's disease and essential tremor, and FDA Breakthrough Device Designation for treatment of brain metastases.

^• ^Device has FDA 510K clearance for ablation of soft-tissues, but is not cleared/approved for BBBD applications.

## Author contributions

Conception and design of the study: CR, CA, WD, RD, and JR. Acquisition of data: BP, BM, E-OC, SC, ML, JNM, YK, JKGM, CA, SP, AM, RS, and JR. Data analysis and interpretation: BP, BM, E-OC, SC, JNM, ML, YK, JG, CA, SP, AM, CR, RD, and JR. Provision of material support for experiments: CA, WD, SP, AM, CR, RD, and JR. Drafting the manuscript: BP, AE, BM, E-OC, SP, and JR. Critical review of the manuscript: CA, SP, RS, CR, and RD. Approval of the final manuscript: BP, AE, BM, E-OC, SC, ML, JNM, YK, JG, CA, SP, AM, RS, CR, WD, RD, and JR. All authors contributed to the article and approved the submitted version.

## Funding

Generation of portions of the data included in this review were supported by grants from the National Institutes of Health (grant numbers R01CA139099, P01CA207206, R01CA213423, R01CA256285, and U01CA224151), the Virginia Biosciences Health Research Corporation, and the Virginia Research Investment Fund.

## Conflict of interest

Authors ML, CA, RD, and JR have issued patents and/or patents pending in the area of irreversible electroporation induced blood-brain barrier disruption and may receive royalties. CR and JR have issued patents for convection enhanced delivery catheter systems and may receive royalties. AM has issued and pending patents in focused ultrasound blood-barrier disruption and may receive royalties.

The remaining authors declare that the research was conducted in the absence of any commercial or financial relationships that could be construed as a potential conflict of interest.

## Publisher's note

All claims expressed in this article are solely those of the authors and do not necessarily represent those of their affiliated organizations, or those of the publisher, the editors and the reviewers. Any product that may be evaluated in this article, or claim that may be made by its manufacturer, is not guaranteed or endorsed by the publisher.
